# Account of a Physical Sign of Pneumonia of the Apex of the Lungs

**Published:** 1847-09

**Authors:** Wm. M. Boling

**Affiliations:** Montgomery, Ala.


*Account of a Physical Sign of Pneumonia of the Apex of the Lungs.* By WM. M. BOLING, M. D., of Montgomery, Ala.—My experience, so far as it extends, is confirmatory of the opinion that pneumonia, commencing at the apex of the lung, is, in proportion to the number of cases, the most frequently fatal form of the disease. I have met with about six cases of this affection, at least have recognized, or identified about that number. They all proved fatal. I will notice three of them :—In one, the subject of which was a powerful and robust Irishman, 30 years old, "fond of a dram," but not decidedly intemperate, and previously in good health,—the disease supervened on an attack of acute bronchitis, about the fifth day, and proved fatal on the fourteenth day, counting from the first of his illness, In the second case, the patient was a rather delicate negro woman about 28 years old,—the attack commenced during a slight indisposition of a catarrhal character, and proved fatal on the thirteenth day. The other patient was a strong and robust negro woman, about 22 years old, previously in good health, and in her case the termination was on the ninth day.

The general symptoms and march of the disease, in these, did not differ in any material point, from those in the more common form of pneumonia, except in the point of commencement, and in this perhaps—that the morbid alteration had proceeded to a less extent, at the time of death, than is commonly the case in the latter ; that is, death supervened from a less extensive local disease. In the other cases, the lung ran most rapidly into a state of hepatization, the solidification not being preceded by the crepitant bronchus, but by a total absence of the respiratory murmur, while the chest over the affected part remained still resonant on percussion.

My object, however, in the present remarks, is simply to speak of a physical sign that was present, in each of the three cases detailed, which I presume also to be present in others of the same character, the observance of which may probably lead to a correct diagnosis at an earlier period, in some instances, than it would otherwise be made. This is a fine mucous or crepitant rhonchus, seemingly seated in the larynx, loud enough to be heard distinctly at the distance of two or three feet from the patient, and so *persistent*, that it is not removable, or but momentarily, by any effort to expectorate which the patient may make, while at the same time there are present none of the signs of bronchitis or laryngitis. Though it is exceedingly annoying to the observer to hear it, because it impresses him with the belief that it is distressing to the patient, and he looks with a feeling rather of impatience for an attempt, by an effort to expectorate, for its removal; the patient seems perfectly indifferent to its presence, which would not be the case were it really produced by the presence of a small quantity of tenacious mucus in the larynx itself. The sound, then, is only seemingly produced in the larynx, for on applying the stethoscope immediately under or just above the clavicles, it will be discovered to proceed from the apex of one or the other lung, which will be found the seat of inflammatory action. It would seem that the sound there produced in the pulmonary vesicles, must be conveyed by the larger bronchial ramifications, numerous and superficial at this point, to the larynx, where, in consequence of the thinness of the tube, or rather the thinness of its covering, and its proximity to the surface, the deceptive impression of its production in this organ, from the presence of a small quantity of viscid mucus, is created.

It is the indifference of the patient to the presence of the sound, but still more especially its *persistence*, which constitutes its peculiar and distinctive feature, and upon which its value as an evidence of pneumonia commencing in the apex of the lung depends. In other affections of the lungs and air passages, more especially in bronchitis, we may have a somewhat similar sound produced in the larynx itself, by the play of the passing air through a small quantity of viscid mucus there collected; but under such circumstances, it is removable by coughing, or an effort to expectorate, and once removed may not return again, or only after a considerable interval, when a fresh collection of mucus has taken place. The patient, too, does not manifest the same indifference in regard to its presence, but the mucus producing it soon excites an effort for its removal.

As pneumonic inflammation, in the greater number of cases, commences at the base of the lung, the inexperienced stethoscopist, on observing the general symptoms of pneumonia present, may neglect to apply his instrument over the apex of the organ in attempting to discover the location and extent of the disease, and failing to detect any physical evidences of morbid action near the base, might at once attribute the symptoms present to inflammation, somewhat circumscribed, of the central portion of the pulmonary texture; too limited in extent, and too remote from the surface, to give rise to the peculiar physical phenomena. To be sure, were he to examine the entire chest, the disease would be detected. The recollection of the sign which I have named, leads at once to its locality.

It is altogether probable, that in a number of instances of this kind, the exact seat of morbid action has escaped my own observation, as the peculiar sign which I have spoken of, I considered indicative of the most extreme danger, long before I discovered its connection with inflammation, commencing at the apex of the lung.—*American Jour. of Med. Sci.*

